# The association of statin therapy and cancer: a meta-analysis

**DOI:** 10.1186/s12944-023-01955-4

**Published:** 2023-11-10

**Authors:** Zijian Chen, Panyun Wu, Jiangang Wang, Pengfei Chen, Zhenfei Fang, Fei Luo

**Affiliations:** 1 Research Institute of Blood Lipid and Atherosclerosis, the Second Xiangya Hospital, Central South University, Changsha, 410011 Hunan China; 2 School of Medicine, Hunan University of Medicine, Huaihua, 418000 Hunan China; 3 Department of Cardiovascular Medicine, The Second Xiangya Hospital, Central South University, Changsha, 410011 Hunan China; 4 Department of Health Management, Central South University, The Third Xiangya Hospital, Changsha, 410013 Hunan China

**Keywords:** Statin, Cancer, Randomized controlled trial, Incidence, Mortality, Meta-analysis, Lipid

## Abstract

**Background:**

Statins are routinely prescribed to lower cholesterol and have been demonstrated to have significant benefits in atherosclerotic cardiovascular disease. However, whether statin therapy has effects on cancer risk remains controversial. In this study, we investigated the influence of statin therapy on cancer incidence and mortality by conducting a comprehensive meta-analysis of randomized controlled trials.

**Methods:**

Systematic searches by Cochrane, Embase, Medline, and PubMed were performed to locate data from eligible randomized controlled trials related to statin therapy and oncology. Our main endpoints were cancer incidence and mortality. Fixed-effects models were used in this study.

**Results:**

This meta-analysis comprised thirty-five randomized controlled studies. Twenty-eight included studies reported cancer incidence, and eighteen reported cancer mortality. The pooled results indicated no reduction in cancer incidence with statins compared to placebo [OR = 0.99, 95% CI (0.95, 1.03)]. In addition, statins did not decrease cancer mortality [OR = 0.99, 95% CI (0.91, 1.07)]. This study also performed a number of subgroup analyses, which showed no effect of statins on cancer subtypes such as genitourinary and breast cancer. Neither the type of statin nor long-term treatment with statins had an effect on cancer incidence and mortality.

**Conclusion:**

Through comprehensive analysis, we found that statin therapy does not reduce cancer incidence or mortality while protecting the cardiovascular system.

**Trial registration:**

Prospero CRD42022377871.

**Supplementary Information:**

The online version contains supplementary material available at 10.1186/s12944-023-01955-4.

## Introduction

In recent years, statins, one of the most efficient cholesterol-lowering drugs, have been widely used, especially for atherosclerotic cardiovascular disease (ASCVD). With many clinical and preclinical studies on statins, the cholesterol-independent or pleiotropic effects of statins are gradually being noted. Studies have found the benefits of statins, including anti-inflammatory effects, reducing oxidative stress, improving endothelial function, and regulating immune reactions [1]. Interestingly, recent preclinical studies have shown that statins have antitumor effects via antiproliferative [2], antiangiogenic [3], and proapoptotic effects [4]. In addition, numerous recent randomized controlled trials (RCTs) have also suggested that statin treatment may be able to reduce cancer incidence and mortality [5–7]. The Justification for the Use of Statins in Prevention: an Intervention Trial Evaluating Rosuvastatin (JUPITER) trial was conducted in 2008 with a sample size of over 10,000 people. The study concluded that rosuvastatin significantly reduced cancer incidence and mortality compared to placebo [7]. According to their findings, the statin therapy group had fewer new cancer diagnoses and fewer cancer deaths than the control group. The trial conducted in 2016, called Heart Outcomes Prevention Evaluation (HOPE)-3, also found that statin treatment decreased tumor incidence [5]. However, some studies suggest that statins may have no significant effect or even increase tumor incidence and mortality [8–10]. The Cholesterol and Recurrent Events (CARE) trial in 1996 [9] and the Pravastatin in elderly individuals at Risk of Vascular Disease (PROSPER) trial in 2002 [10] indicated a notable rise in the incidence and mortality of cancer among patients taking pravastatin compared to those taking placebo. Both of these studies enrolled at least 4,000 individuals at an average age of 67 years and followed them for more than 3 years [9, 10]. Krista and colleagues published a meta-analysis in 2006 that showed statins did not affect cancer incidence and mortality [11]. However, this study did not include some important studies with larger sample sizes, such as JUPITER [7] (*n* = 17,802) and (HOPE)-3 [5] (*n* = 12,705), which reported that statins could reduce the incidence of cancer. After this meta-analysis, there were many more RCTs examining the relationship between statin treatment and tumors. Statins can benefit individuals with ASCVD, as evidenced by researchers. However, existing research, including clinical studies and meta-analyses, is controversial as to whether statins have an effect on cancer incidence. Several studies support the assumption that statins have a positive impact on reducing the incidence of cancer. Recent preclinical studies have also found that statins may inhibit the proliferation of cancer cells. We hypothesized that statins might have beneficial results in reducing cancer incidence and mortality. Our article further clarifies the relationship between statin treatment and tumor incidence by meta-analyzing all relevant RCTs from 1900 to the present. This provides a valuable reference for clinicians and statin users.

## Methods

During the writing of this meta-analysis, all our procedures strictly followed the guidelines in the Preferred Reporting Items for Systematic Reviews and Meta-Analyses (PRISMA) statement [12]. A version of the protocol at the beginning of this meta-analysis study was approved and filed with the International Prospective Registry for Systematic Reviews (PROSPERO). [No: CRD42022377871].

### Search strategy

The following four databases, MEDLINE, EMBASE, COCHRANE, and PUBMED, were utilized to conduct a comprehensive and systematic literature search from 1 January 1900 to 1 September 2023, restricted to studies of humans published in the English language. The following MeSH terms and combined text were leveraged for searching: "Hydroxymethylglutaryl-CoA Reductase Inhibitors" and "randomized controlled trial". The complete search equation for PubMed was ((((Hydroxymethylglutaryl-CoA Reductase Inhibitors) OR (Hydroxymethylglutaryl-CoA Reductase Inhibitors[MeSH Terms])) OR (Statin[Text Word])) AND (randomized controlled trial)) AND (("1900/01/01"[Date—Publication]: "2023/09/01"[Date—Publication])). We considered all studies that might be eligible for this meta-analysis, regardless of their time constraints or primary outcome. Endnote X9 was used for citation management.

### Eligibility criteria

Studies were deemed suitable for inclusion if they were prospective randomized clinical trials of statins and contained indicators of cardiovascular events, had a comparison of statin treatment and nonstatin controls, and reported data on cancer incidence or cancer mortality. The three categories of placebo, conventional therapy, and no therapy were considered nonstatin controls. Statin therapies included monotherapy or add-on therapies to conventional therapies. Observational and retrospective studies, comparisons between different types and dosages of statins, and studies without definitive cancer incidence or mortality were excluded. The number of participants and study duration were not limited to avoid excluding or losing any studies with cancer incidence or mortality.

### Study selection and data extraction

The search for the entire study was conducted independently by two investigators (ZC and PW). They finalized the research that satisfied the eligibility criteria for inclusion by screening the titles and abstracts to obtain literature that met the initial screening and then performing a secondary screening by reading the full text. Discrepancies were settled with consensus.

Two of us independently piloted a data collection form and separately extracted outcome data of trials selected for detailed analysis. Extracted data were compared by the third author, and any discrepancies were resolved through discussion. We attempted to contact study authors if data were not available.

In each of the final chosen studies, the following data were extracted: authorship, publication year, study area, type of study design (randomized or not, with or without control and kind of control, with or without blinding and type of blinding), sample size and sex ratio of included population, participant follow-up duration, statin treatment (dose, type), lipid levels before and after the test, and specific types of cancer diagnosed (respiratory, breast, skin papilloma, melanoma, connective tissue, prostate, lymphoma, pancreas, bladder, gastrointestinal, or hematological). If a search revealed that more than one study had been published in the same population or patient group, we tended to select the first published study. If the desired outcome data did not appear in the first published study, then the later studies were selected.

### Risk of bias assessment

The included studies' risk of bias and quality of evidence were independently examined and assessed by two researchers (PW and FL). To estimate the risk of bias, we have mastered the essentials of risk assessment and are proficient in the application of the Cochrane Risk of Bias Tool. The included RCTs were evaluated in multiple dimensions, such as randomization, random assignment concealment, treatment assignment masking, and blinding. To prevent discrimination from naked eye estimation, we used the Egger regression test to quantitatively assess possible publication bias. Discrepancies were resolved by discussion. If information was not reported in the article and/or clarification was needed, we contacted the author.

### Data synthesis and analysis

Cancer incidence and mortality were considered dichotomous variables. We used fixed-effects models (Mantel-Haunzel method) with calculated weighted means reported as odds ratios (ORs). Meanwhile, the parameter range was estimated using 95% confidence intervals (CIs).

The Cochran Q test plays an essential role in research and is used to assess the heterogeneity between studies (*P* < 0.05 represents statistical significance). In addition, the I^2^ test played a vital role in further evaluating the heterogeneity among the incorporated RCTs. If the resulting value of I^2^ is greater than 50%, it indicates a moderate or even high degree of heterogeneity. RevMan (version 11.0) and Stata (version 17.0) played an integral part in performing the statistical analysis.

In the presence of no considerable heterogeneity (Q-test *P* > 0.05 and I^2^ < 50%), summary ORs and 95% CIs were computed with a fixed-effects model. After detecting publication bias, we ran sensitivity analyses and checked the robustness of the overall results by removing each paper individually.

## Results

### Search results and baseline characteristics of studies

**Fig. 1:**
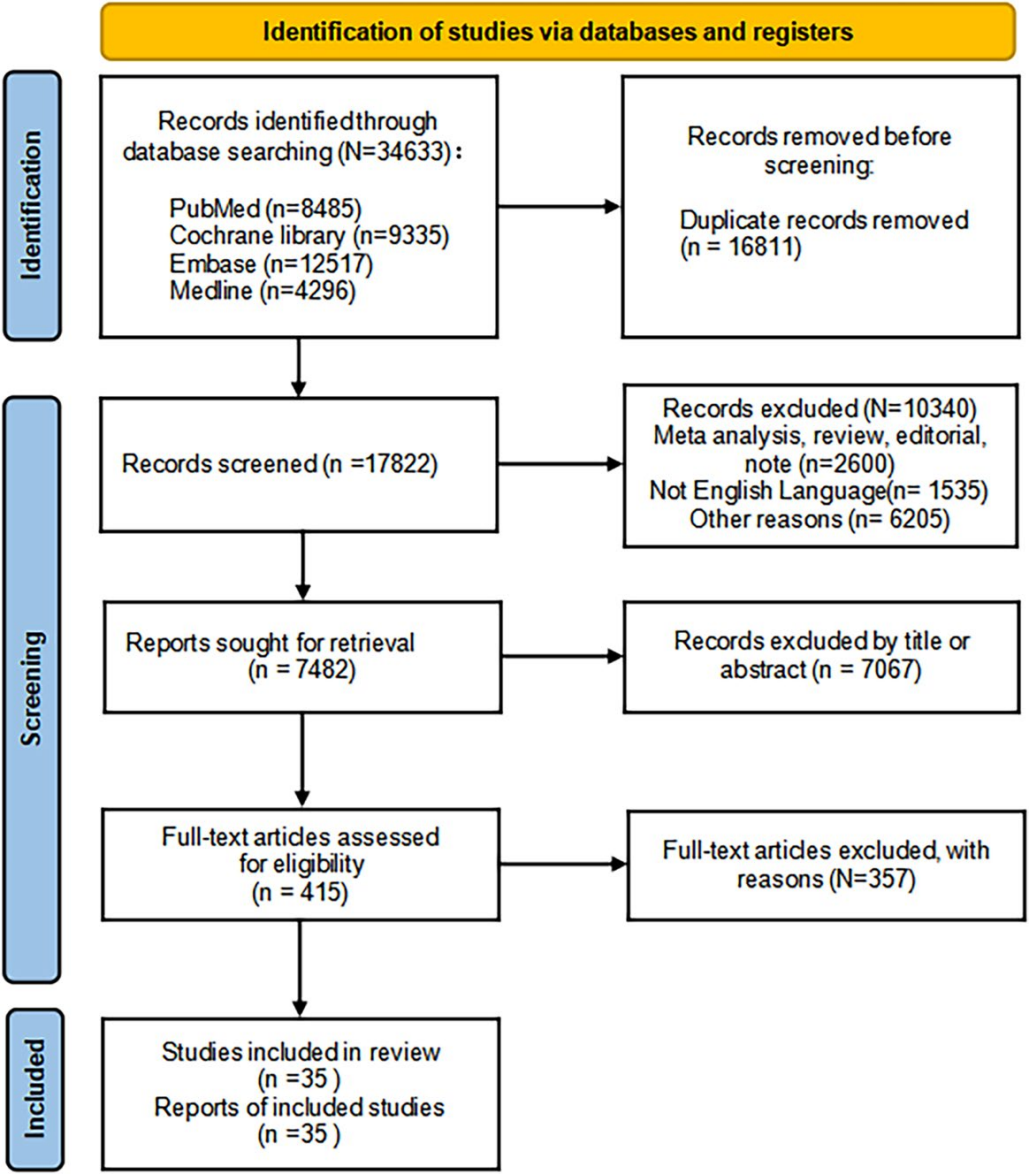
Flow chart of study selection in the present meta-analysis

The database search flowchart is shown in Fig. 1. Automated software detects retracted and duplicate reports and removes them from the screening process. We identified 17,822 possible studies after an initial search and screening for duplicates (Fig. 1). Humans, English language, and clinical trials were considered limitations in the search, and studies were scrutinized, ultimately eliminating 10,340 of them. In addition, 7482 articles' abstracts and titles were examined, and 7067 further articles were disregarded. A complete and thorough reading of the full text of the remaining 415 studies excluded 357 studies that did not fall within the eligibility criteria. Eventually, 35 studies that reported tumor incidence or mortality were included. Of these 35 studies, 28 provided information on the incidence of cancer, while a total of 18 studies reported data on cancer mortality. Baseline data and other parameters of the included RCTs are presented in Table 1.

**Table 1:** Characteristics of studies included in the meta-analysis

Study	Name of study	Area	Study design	Median follow–up, y	Treatments	Sample size		Gender (female, %)	
						**statin**	**non statin**	**statin**	**non statin**
Blankenhorn et al., 1993 [13]	MARS	NR	R, DB, PC	3.7	lovastatin (80 mg daily) or placebo	123	124	NR	NR
Terje et al., 1994 [14]	4S	Scandinavia	R, DB, PC	5.4	simvastatin(10–40 mg daily) or placebo	2221	2223	407(18%)	420(19%)
Oliver et al., 1994 [15]	MAAS	Europe	R, B, PC	4	simvastatin (20 mg daily) or placebo	193	188	22(11%)	23(12%)
Wouter et al., 1995 [16]	REGRESS	Netherlands	R,DB, PC	2	Pravastatin or placeo	450	434	0(0%)	0(0%)
Shepherd et al., 1995 [17]	NR	the West of Scotland district	R, DB, PC	4.9^a^	pravastatin (40 mg daliy) or placebo	3302	3293	0(0%)	0(0%)
Frank et al., 1996 [9]	CARE	Canada, the United States	R, DB, PC	5	pravastatin (40 mg daliy) or placebo	2081	2078	291(14%)	291(14%)
Alan et al., 1997 [18]	LCAS	the United States	R, DB, PC	2.5	fluvastatin (20 mg twice daily) or placebo	214	215	48(22.4%)	32(14.9%)
Bestehorn et al., 1997 [19]	CIS	Germany	R, DB, PC	2.3^a^	simvastatin (20–40 mg) or placebo	129	125	0(0%)	0(0%)
Tonkin et al., 1998 [20]	LIPID	Australia, Zealand	R, DB, PC	6.1^a^	pravastatin (40 mg daily) or placebo	4512	4502	756(17%)	760(17%)
John et al., 1998 [21]	AFCAPS/TexCAPS	Texas	R, DB, PC	5.2^a^	Lovastatin (20–40 mg daily) or placebo	3304	3301	499(15%)	498(15%)
Jun et al., 2000 [22]	KLIS	Japan	R, PC	5^a^	pravastatin (10–20 mg daily) or conventional treatment	2219	1634	0(0%)	0(0%)
Teo et al., 2000 [23]	SCAT	Canada	R, DB, PC	4	simvastatin or placebo	230	230	29(13%)	21(9%)
John et al., 2002 [24]	LIPID follow-up	Australia and New Zealand	R, DB, PC	6	pravastatin (40 mg daily) or placebo	4512	4502	661(17%)	658(17%)
Shepherd et al., 2002 [10]	PROSPER	Scotland, Ireland, and the Netherlands	R, B, PC	3.2^a^	pravastatin (40 mg daily) or placebo	2891	2913	1495 (51.7%)	1505 (51.7%)
Jeffrey et al., 2002 [25]	ALLHAT-LLT	United States, Puerto Rico, US Virgin Islands, and Canada	R, PC	4.8	Pravastatin (40 mg daily) or usual care	5170	5185	2511 (48.6%)	2540(49%)
Serruys et al., 2002 [26]	LIPS	Belgium, France, Germany, Italy, United Kingdom, the Netherlands, Spain, Switzerland, Canada, and Brazil	R, DB, PC	3.9	fluvastatin (80 mg daily) or placebo	844	833	133(15.8%)	138 (16.6%)
Heart Protection Study Collaborative Group, 2002 [27]	HPS	UK	R, PC	5	simvastatin (40 mg daily) or placebo	10,269	10,267	8421(82%)	1643(16%)
Hallvard et al., 2003 [28]	ALERT	Belgium, Denmark, Finland, Germany, Norway, Sweden, Switzerland, the UK, and Canada	R, DB, PC	5.1^a^	fluvastatin (40 mg daily) placebo	1050	1052	349(33.2%)	366 (34.8%)
Peter et al., 2003 [29]	ASCOT-LLA	Nordic countries, UK and Ireland	R, DB, PC	3.3^a^	atorvastatin (10 mg daily) or placebo	5168	5137	979(18.9%)	963 (18.7%)
Beishuizen et al., 2004 [30]	NR	the Hague	R, DB, PC	2	cerivastatin(0.4 mg daily) or placebo	125	125	64(51%)	68(54%)
Helen et al., 2004 [31]	CARDS	UK and Ireland	R, PC	3.9	atorvastatin (10 mg daily) or placebo	1428	1410	456(32%)	453(32%)
Michael et al., 2004 [32]	The ALLIANCE Study	America	R	4.3	atorvastatin or usual care	1217	1225	217(17.8%)	217 (17.7%)
Wanner et al., 2005 [33]	4D	Germany	R, DB, PC	4	atorvastatin (10 or 20 mg daily) or placebo	619	636	286(46.2%)	292 (45.9%)
Stegmayr et al., 2005 [8]	NR	NR	R, PC	2.6^a^	atorvastatin or placebo	70	73	22(31.4%)	21(30.1%)
Nakamura et al., 2006 [6]	MEGA Study	JAPAN	R, PC	5.3^a^	diet plus 10–20 mg pravastatin daily or diet	3866	3966	2638(68%)	2718(69%)
Pierre et al., 2006 [34]	SPARCL	NR	R, DB, PC	4.9	atorvastatin (80 mg daily) or placebo	2365	2366	938(39.7%)	970 (41.0%)
Kjekshus et al., 2007 [35]	CORONA	19 European countries, Russia, and South Africa	R, B, PC	2.7	rosuvastatin (10 mg daily) or placebo	2541	2479	593 (24%)	587(24%)
Paul et al., 2008 [7]	JUPITER	1315 sites in 26 countries	R, DB, PC	1.9	rosuvastatin (20 mg daily) or placebo	8901	8901	3426 (38.5%)	3375 (37.9%)
Kwan et al., 2010 [36]	ASTRONOMER	Canada	R, DB, PC	3.5	rosuvastatin (40 mg daily) or placebo	134	135	53 (39.5%)	50(37%)
Bengt et al., 2010 [37]	AURORA	280 centers in 25 countries	R, DB, PC	3.8	rosuvastatin (10 mg daily) or placebo	1389	1384	538 (38.7%)	512(37%)
Schanberg et al., 2011 [38]	APPLE	North American	R, DB, PC	3	atorvastatin (10 or 20 mg daily) or placebo	113	108	95 (84.1%)	89(82.4%)
Hosomi et al., 2015 [39]	J-STARS	Japan	PROBE^b^	4.9	pravastatin (10 mg daily) or no statins	793	785	248 (31.3%)	243(31%)
Yusuf et al., 2016 [5]	(HOPE)–3	228 centers in 21 countries	R, DB, PC	5.6	rosuvastatin (10 mg daily) or placebo	6361	6344	2951 (46.4%)	2923 (46.1%)
Kitas et al., 2019 [40]	TRACE RA	UK	R, DB, PC	2.5	atorvastatin (40 mg daily) or placebo	1504	1498	1120 (74.8%)	1107 (73.6%)
Wang et al., 2021 [41]	OAKS	Australia	R, DB, PC	2	atorvastatin (40 mg daily) or placebo	151	153	92 (60.9%)	77(50.3%)

^a^Studies are presented as the mean follow-up

^b^PROBE means prospective randomized, open labeled, blinded-endpoint

*Abbreviations*: *R* randomized, *DB* double-blinded, *PC* placebo-controlled, *NR* not reported, *MARS* Monitored Atherosclerosis Regression Study, *4S* Scandinavian Simvastatin Survival Study, *MAAS* Multicenter Anti-Atheroma Study, *REGRESS* Regression Growth Evaluation Statin Study, *CARE* Cholesterol and Recurrent Events, *LCAS* Lipoprotein and Coronary Atherosclerosis Study, *CIS* Multicenter Coronary Intervention Study, *LIPID* Long-Term Intervention With Pravastatin In Ischemic Disease, *AFCAPS/TexCAPS* Air Force/Texas Coronary Atherosclerosis Prevention Study, *KLIS* Kyushu Lipid Intervention Study, *SCAT* The Simvastatin/Enalapril Coronary Atherosclerosis Trial, *PROSPER* Pravastatin In Elderly Individuals At Risk Of Vascular Disease, *ALLHAT-LLT* Antihypertensive and Lipid-Lowering Treatment to Prevent Heart Attack Trial, *LIPS* Lescol Intervention Prevention Study, *HPS* Heart Protection Study, *ALERT* Assessment of LEscol in Renal Transplantation, *ASCOT-LLA* Anglo-Scandinavian Cardiac Outcomes Trial—Lipid Lowering Arm, *CARDS* Collaborative Atorvastatin Diabetes Study, *ALLIANCE* Aggressive Lipid-Lowering Initiation Abates New Cardiac Events, *MEGA* Management of Elevated Cholesterol in the Primary Prevention Group of Adult Japanese, *SPARCL* Stroke Prevention by Aggressive Reduction in Cholesterol Levels, *CORONA* Controlled Rosuvastatin Multinational Trial in Heart Failure, *JUPITER* Justification for the Use of Statins in Prevention: an Intervention Trial Evaluating Rosuvastatin, *ASTRONOMER* Aortic Stenosis Progression Observation: Measuring Effects of Rosuvastatin, *AURORA* A Study to Evaluate the Use of Rosuvastatin in Subjects on Regular Hemodialysis: An Assessment of Survival and Cardiovascular Events, *APPLE* Atherosclerosis Prevention in Pediatric Lupus Erythematosus, J-*STARS* The Japan Statin Treatment Against Recurrent Stroke, HOPE Heart Outcomes Prevention Evaluation, *TRACE RA* the Trial of Atorvastatin for the Primary Prevention of Cardiovascular Events in Patients with Rheumatoid Arthritis, *OAKS* The Osteoarthritis of the Knee Statin

### Results from the quantitative synthesis

**Fig. 2:**
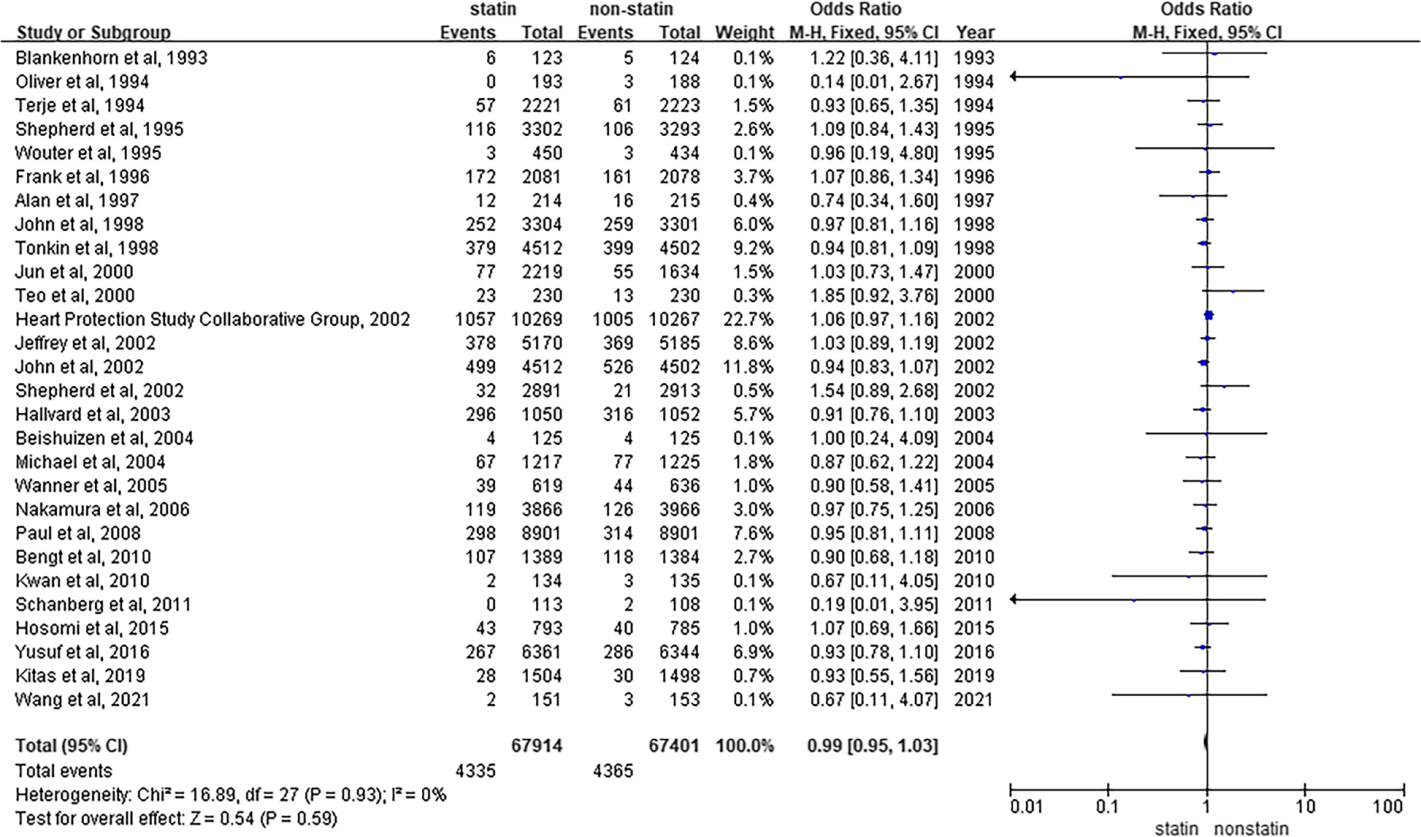
Overall meta-analysis of statin use and cancer incidence

To evaluate the risk of cancer development in patients who were receiving statins, we summarized and analyzed 28 RCTs that included cancer incidence using a fixed-effects model. Compared to those who did not use statins, we found that regular statin therapy for a period of time did not result in a substantial reduction in tumor incidence [OR = 0.99, 95% CI (0.95, 1.03), *P* = 0.59] (Fig. 2). Moreover, by analyzing and summarizing the collected data from the included studies, we concluded that the heterogeneity among the 28 studies mentioned above was low (I^2^ = 0%).

**Fig. 3:**
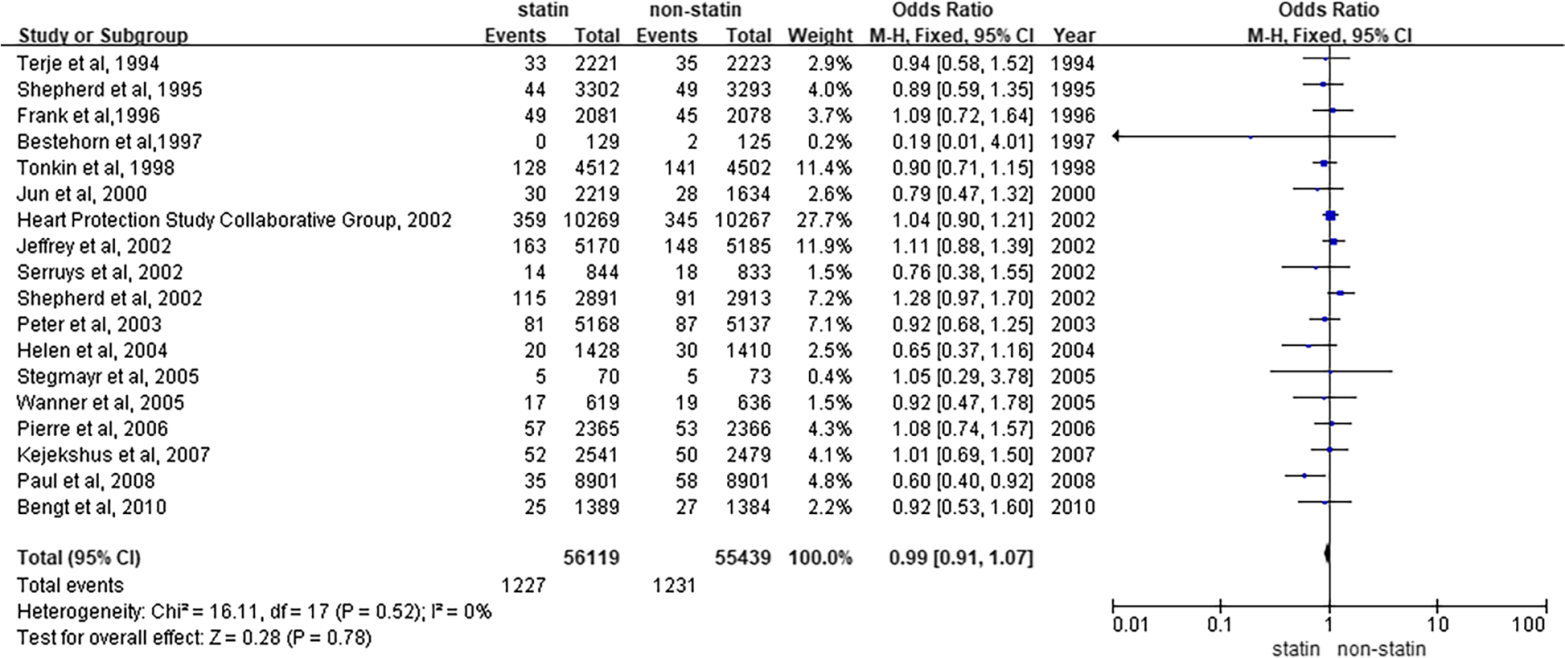
Overall meta-analysis of statin use and cancer mortality

To explore the risk of tumor mortality associated with a period of statin therapy, we conducted an analysis of data from 18 studies using a fixed-effects model. Similarly, compared to nonstatin controls, we found that there was no noticeable reduction in cancer mortality after a period of treatment with statins in patients [OR = 0.99, 95% CI (0.91, 1.07), *P* = 0.78] (Fig. 3). In addition, the forest plot shows very low heterogeneity between the above 18 studies (I^2^ = 0%). In the subgroup analysis, we selected several typical cancers for analysis. The results are as follows: genitourinary [OR = 0.99, 95% CI (0.86, 1.44), *P* = 0.92], breast [OR = 1.13, 95% CI (0.81, 1.59), *P* = 0.47), respiratory [OR = 0.97, 95% CI (0.83, 1.15), *P* = 0.74] and gastrointestinal cancer [OR = 1.01, 95% CI (0.88, 1.17), *P* = 0.84]. The combined results revealed that statin use had no reducing impact on the incidence of several of these typical cancer subtypes (see supplemental Fig. 1).

We divided the statins used in the included studies into hydrophilic and lipophilic statins and compared their effects on cancer incidence and mortality. The results showed that neither hydrophilic nor lipophilic statins affected cancer incidence and mortality (see supplemental Fig. 2 and 3).

Statins generally need to be taken for long periods of time or even for whole life. To explore the influence of prolonged statin treatment on tumor incidence and mortality, we analyzed studies in which the duration of statin use was more extended than or equal to 4 years, as well as those with shorter than 4 years of follow-up, and both obtained the same results as the above subgroup analysis (see supplemental Fig. 2 and 3).

Meanwhile, the risk of stroke, cardiovascular event mortality, cardiovascular event rate, and myocardial infarction were also analyzed. Our findings indicate a noticeable risk decrease in stroke [OR = 0.80, 95% CI (0.76, 0.86), *P* < 0.00001], cardiovascular event mortality [OR = 0.83, 95% CI (0.79, 0.87), *P* < 0.00001], cardiovascular event rate [OR = 0.80, 95% CI (0.77, 0.83), *P* < 0.00001], and myocardial infarction [OR = 0.68, 95% CI (0.65, 0.72), *P* < 0.00001] among statin users compared with nonstatin users (see online supplemental Fig. 4).

To estimate whether the overall impact size of statins on cancer changed over time and whether heterogeneity existed, we removed each study separately for further sensitivity analysis. Following a thorough sensitivity analysis, the overall effect magnitude and heterogeneity remained unchanged (data not shown).

### Quality appraisal

**Fig. 4:**
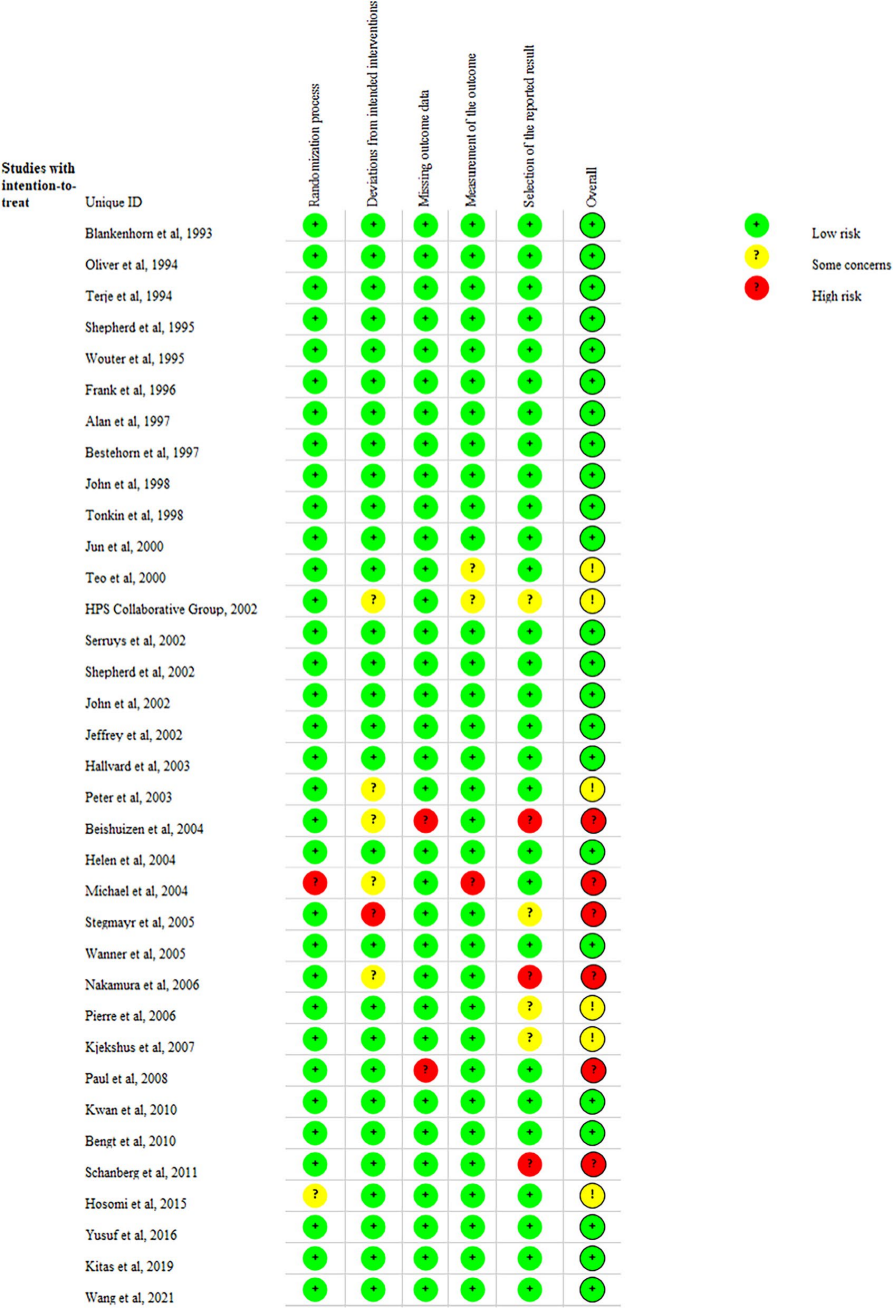
Traffic light plot showing the risk of bias assessment based on the authors' opinion of each risk area described in the RoB2 tool for the included RCTs

**Fig. 5:**
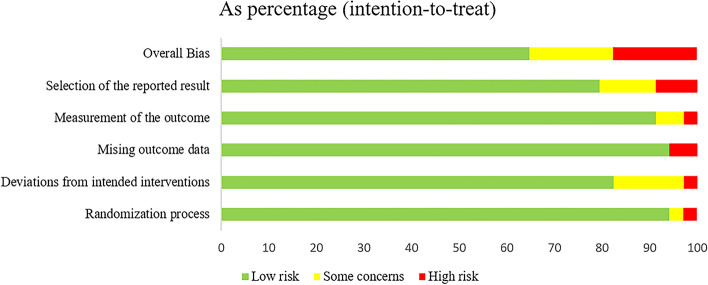
A summary plot of the risk of bias assessment is displayed based on the authors' opinion of each risk area described in the RoB2 tool for the included RCTs

We evaluated the final included studies in accordance with the methodology specified in the review protocol. The RoB2 tool was used to assess RCTs. The traffic light diagram (Fig. 4) and the summary diagram (Fig. 5) graphically and visually reflect the risk of bias estimates for the chosen RCTs in each risk area. Of the 35 studies, the majority (*n* = 23) were judged as being at low risk of bias, and a proportion (*n* = 6) were assessed as unclear for reasons such as deviation from intended intervention. Selectivity in reporting results was the primary source of risk of bias. The studies by Yusuf et al*.* in 2016 [5] and Bengt et al*.* in 2010 [37] were considered to have the lowest risk of bias.

### Publication bias

**Fig. 6:**
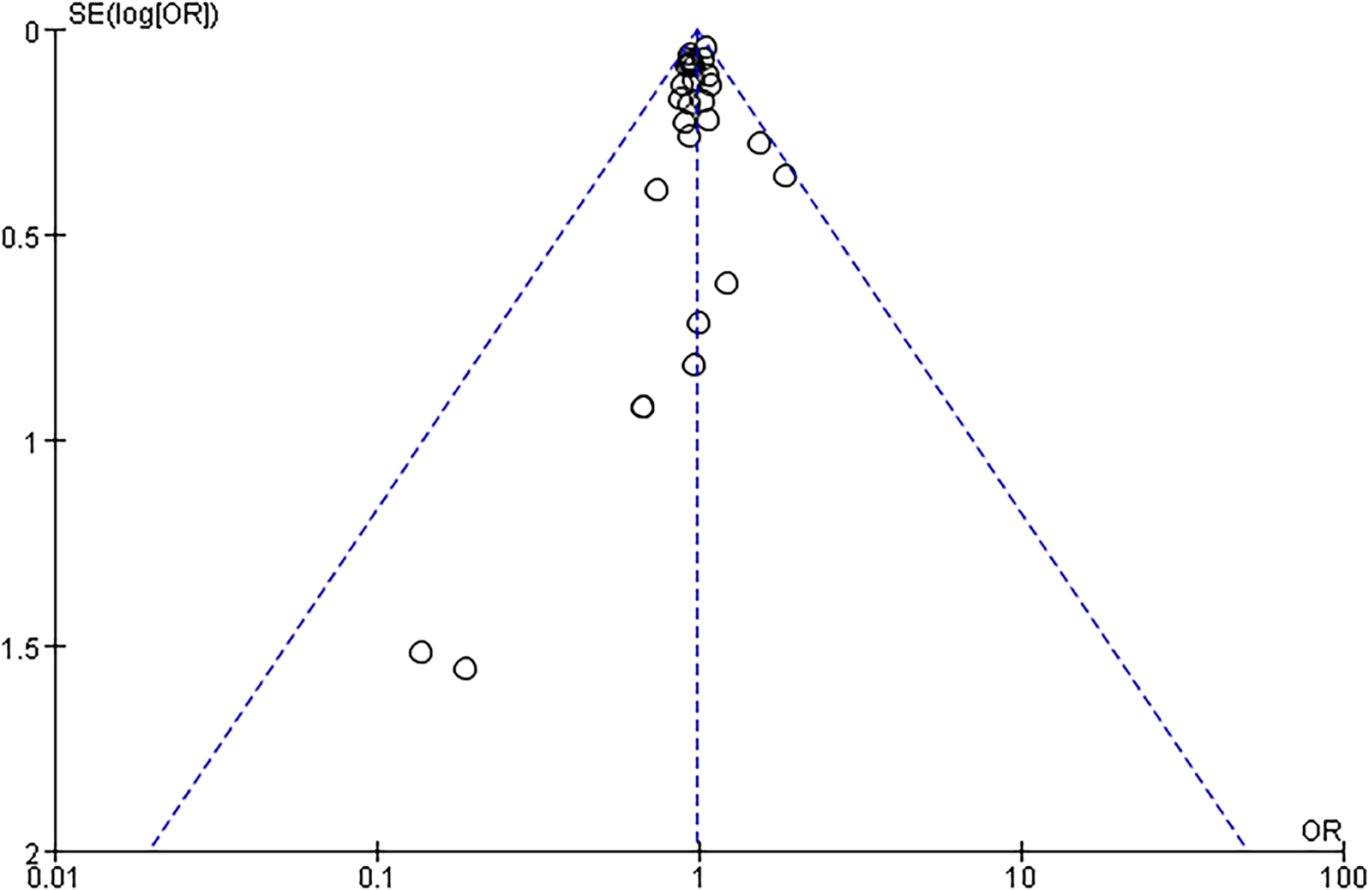
Funnel plots represent data from 28 studies of cancer incidence

**Fig. 7:**
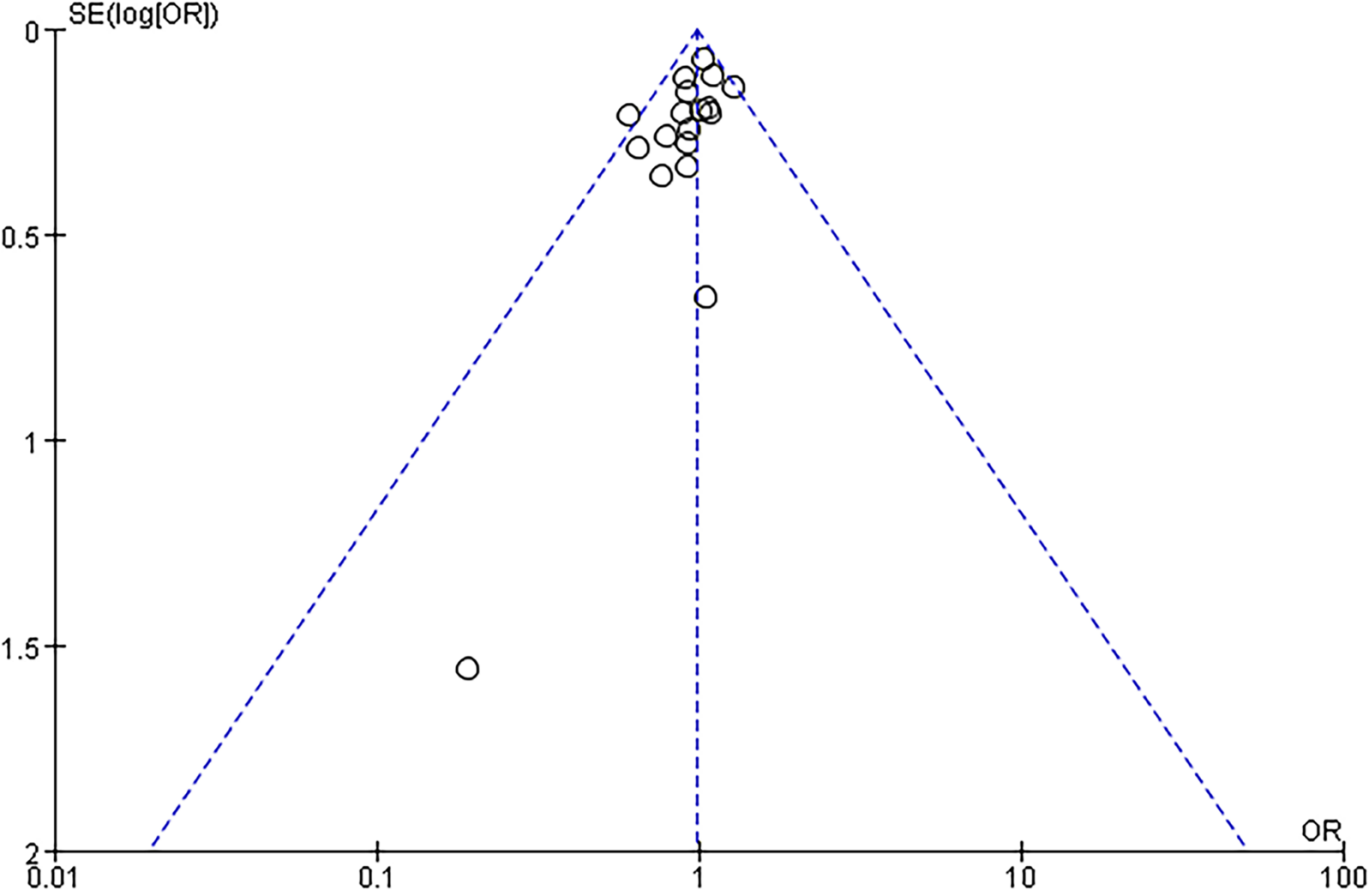
Funnel plots represent data from 18 studies of cancer mortality

Figures 6 and 7 show funnel plots of statin treatment versus placebo/control treatment in terms of cancer incidence and cancer mortality, respectively. In the present meta-analysis, the publication bias is marginally asymmetric but difficult to quantify. Therefore, we performed further tests. The outcome of Begg's test for cancer incidence (*P* = 0.650) indicated that there was no latent publication bias, as evidenced by Egger's test (*P* = 0.357). Whereas the findings of Begg's test for cancer mortality (*P* = 0.034) indicated the existence of publication bias, further Egger's test reached the same conclusion (*P* = 0.034).

## Discussion

The connection between statin treatment and cancer risk was assessed in our current meta-analysis. The current study suggested that statin therapy does not decrease cancer incidence or mortality while protecting the cardiovascular system and reducing the incidence of ASCVD.

Basic and clinical research suggests that statins may be beneficial in triple-negative breast cancer (TNBC) [42]. Furthermore, there have been numerous meta-analyses indicating that statins have a positive impact on biliary tract cancers [43] and gastric cancer [44]. It is possible that statins may affect the incidence and mortality of some specific cancers. Therefore, we performed a subclass analysis on different types of cancer and statin treatment. The results of the study suggested that statins had no apparent influence on the incidence of genitourinary cancer, breast cancer, gastrointestinal cancer, or respiratory cancer.

Statins are defined as either lipophilic or hydrophilic based on their solubility in water or lipid-containing environments. They both have distinct pharmacologic effects. It is generally known that lipophilic and hydrophilic statins play distinct roles in cancer prevention [45]. Zhang and his colleagues analyzed observational studies and concluded that exposure to both lipophilic statins and hydrophilic statins reduced the incidence of hepatocellular carcinoma compared to the unexposed cohort [46]. In our study, through analysis and summarization, we found that neither lipophilic statins nor hydrophilic statins reduced cancer incidence and mortality. However, we suppose that there are several factors contributing to the difference in results through careful comparison and analysis. First, the types of studies included in the analysis are different. Zhang's study used all observational studies, whereas we included RCTs. Second, their article only focused on liver cancer. In our study, there were insufficient data on liver cancer for analysis. Last, in terms of research methods, our study used different data analysis software, which may also affect the results.

In clinical practice, statin users generally need to take statins for long periods of time and even for a lifetime in some cases. Therefore, it is possible that the duration of statin therapy may have an impact on cancer incidence or mortality. To assess the potential duration effect, the included studies were divided into two groups according to the duration of follow-up; those with a treatment duration longer than or equal to 4 years were classified as one group, and the other group was shorter than 4 years. The data of the two groups were analyzed and summarized separately. The results shown in the forest plot indicate that the duration of statin therapy did not have a remarkable influence on cancer incidence and mortality.

Statins are widely recognized as both safe and effective in preventing ASCVD. For the relationship between statin therapy and cancer, the results of some studies are similar to ours. Based on analyzing and summarizing observational studies, they concluded that statins are not directly related to the development of cancer [47, 48]. Some studies have come to different conclusions from ours, demonstrating that treatment with statins for a period of time can be effective in lowering the incidence of cancer and that it may even be possible to use statins for cancer treatment. A summary analysis of included case‒control studies, cohort studies, and RCTs by Livia and her colleagues revealed that statin treatment decreased the overall risk of pancreatic cancer by 30% [49]. It is hypothesized that the reason for the different conclusions may be the different types of studies included in the analysis. RCTs are of great value in clinical research and are recognized as the gold standard for evaluating interventions. It can provide convincing evidence of the effect of a study treatment on human health. We intended to demonstrate the connection between statin treatment and tumor risk in the simplest and intuitively possible manner.

### Strengths and limitations

There are some advantages to this study. First, it is the most recent meta-analysis assessing the connection between statin treatment and tumor risk. To summarize overall effect sizes and to ensure that the greatest available evidence was obtained via meta-analysis methods, we included only RCTs. Second, the sample size of the meta-analysis was substantial, and through analysis, the RCTs included in this study were of high quality. Third, as we accounted for potential confounders in estimating the pooled summary size, statin-related factors could be identified as a single variable affecting cancer in our study. In addition, heterogeneity among the studies incorporated into the analysis was low, and publication bias across studies was relatively small, suggesting that the conclusions drawn from the studies are highly credible. In addition to the data analysis, we have summarized some of the relevant basic research to further demonstrate the relationship between statin treatment and tumors, which may provide some clues and ideas for future research.

This study also has several limitations. First, there is limited information about the types and indications of statins; therefore, we cannot summarize effect sizes based on dose and individual statin types. Second, some RCTs have population limitations, such as the study by Beishuizen and his colleagues that limited the inclusion of the population to people with diabetes, which differs from most of the studies and may result in some bias. Third, although the overall sample we included in the study was large, with relatively high overall tumor incidence and mortality rates, the small sample sizes for analysis of individual specific tumors made it challenging to derive statistically representative results. In addition, the types of statins used in the RCTs we included were different, as were the doses. In clinical practice, different statins have the same efficacy at different dosages, so it is difficult to compare and analyze them purely on the basis of dosage figures. In addition, we cannot rule out that some RCTs do not publish negative results on cancer mortality, which may be a source of publication bias. In future studies, all possible risk factors should be considered, and combined effect sizes should be used to demonstrate a closer connection between statin therapy and cancer risk.

## Conclusion

In summary, our analysis suggests that taking statins to prevent or treat cardiovascular events does not decrease cancer incidence and mortality. Therefore, statins have no protective effect against cancer. However, we have found that statins do not increase cancer risk, so there is no need to worry about an elevated risk of cancer when using them. Studies containing more patients over extended periods are needed to validate our findings further.

### Supplementary Information

**Additional file 1: Supplemental figure 1.** Risk of specific cancer incidence associated with statins.** Supplemental figure 2.** Risk of cancer incidence associated with different types of stains and duration of statin treatment.** Supplemental figure 3.** Risk of cancer mortality associated with different types of stains and duration of statin treatment.** Supplemental figure 4.** Overall meta-analysis of statin use and Cardiovascular disease.** Supplementary Table. **Search strategy of English database. PRISMA 2009 Checklist.

## Data Availability

Data are accessible with a valid request. Please contact the corresponding author if necessary.

## Abbreviations

ASCVD: Arteriosclerotic cardiovascular disease
RCT: Randomized controlled trial
OR: Odds ratio
CI: Confidence interval
TNBC: Triple-negative breast cancer
JUPITER: Justification for the Use of Statins in Prevention: an Intervention Trial Evaluating Rosuvastatin
HOPE: Heart Outcomes Prevention Evaluation
CARE: Cholesterol and Recurrent Events
PROSPER: Pravastatin in elderly individuals at risk of vascular disease
PRISMA: Preferred Reporting Items for Systematic Reviews and Meta-Analyses
